# Supplementary material for: Multikinase Treatment of Glioblastoma: Evaluating the Rationale for Regorafenib
Source: Cancers (Basel). 2025 Jan 23;17(3):375. doi: 10.3390/cancers17030375 (PMC11816343; doi:10.3390/cancers17030375)
Supplement: Supplementary file 1 [file cancers-17-00375-s001.zip › cancers-3382881-supplementary.pdf]

cancers

MDPI

Multikinase Treatment of Glioblastoma: Evaluating the Rationale for Regorafenib

Ana Mª Muñoz-Marmol, Bárbara Melendez, Ainhoa Hernandez, Carolina Sanz, Marta Domenech, Oriol Arpí -Llucía, Marta Gut, Anna Esteve, Anna Esteve-Codina, Genis Parra, Cristina Carrato, Iban Aldecoa, Mar Mallo, Estela Pineda, Francesc Alameda, Nuria de la Iglesia, Eva Martinez-Balibrea, Anna Martinez-Cardús, Anna Estival and Carmen Balana

Supplementary Data

Supplementary Table S1: Patient characteristics

Supplementary Table S2. Excel sheet showing the results of the functional enrichment analysis. (https://biit.cs.ut.ee/gprofiler/gost). Of the 46 genes studied, 19 were found to be involved in angiogenesis pathways.

Supplementary Table S3. Excel sheet showing mutations detected in our series of 103 newly diagnosed GBM patients. Mutations were classified according to their oncogenic potential based on the recommendations of Clinical Genome Resource (ClinGen), Cancer Genomics Consortium (CGC), and Variant Interpretation for Cancer Consortium recommendations.

Supplementary Figure S1. Correlation of copy number variants (CNV) with gene expression by RNA-Seq.

Supplementary Figure S2. Venn diagram showing the overlap between the 18 genes encoding PKs targetable by regorafenib at clinically achievable concentrations and the 19 genes involved in angiogenesis.

Supplementary Table 1. Patient characteristics

Characteristic	All patients (N=103)
**Sex**	
**Male**	56 (54.4%)
**Female**	47 (45.6%)
**Age, yrs – median (range)**	62 (33-80)
**≤65**	66 (64.1%)
**>65**	37 (35.9%)
**KPS**	
**≥70%**	94 (91.3%)
**<70%**	9 (8.7%)
**Extent of surgery**	
**Gross total resection**	26 (25.2%)
**Subtotal/biopsy**	77 (74.7%)
***MGMTp* status**	
**Methylated**	47 (45.6%)
**Unmethylated**	54 (52.4%)
**Unknown**	2 (1.9%)
IDH* mutation (*IDH1*)	
**Mutated**	3 (2.9%)
**Wild-type**	100 (97.1%)
***TERTp* mutation**	
**C228T**	57 (55.3%)
**C250T**	18 (17.5%)
**Wild-type**	20 (19.4%)
**Unknown**	8 (7.8%)
***EGFR* amplified**	46 (44.7%)

- No patient had an IDH2 mutation.
- No patient had a H3 G34 or H3.3 K27 mutation.

Figure S1. Correlation of copy number variants (CNV) with gene expression by RNA-Seq. Informative results for both analyses were available for 71 patient samples.

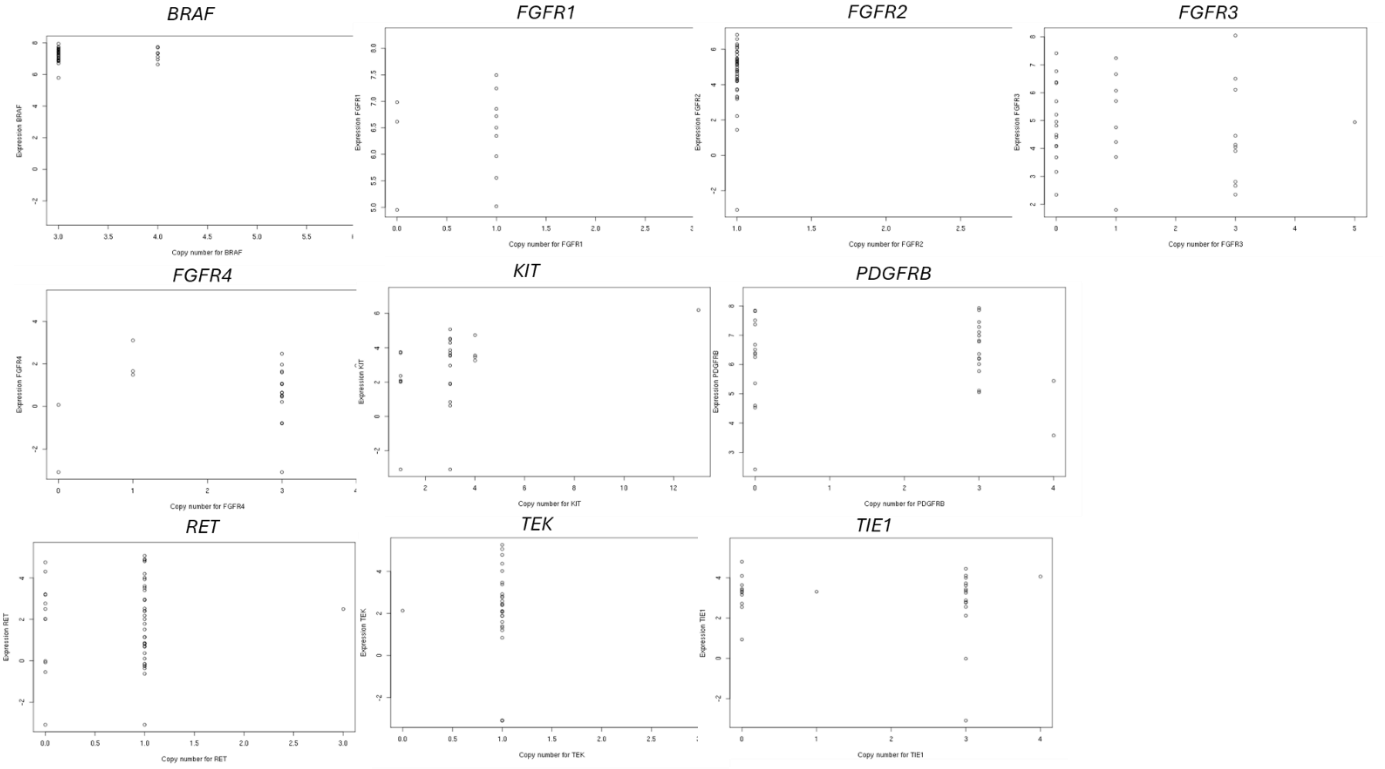

Figure S2. Venn diagram showing the overlap between the 18 genes encoding PKs targetable by regorafenib at clinically achievable concentrations (based on data from DrugBank, PubChem, the EMA and the FDA) and the 19 genes involved in angiogenesis (based on results of our functional enrichment analysis of 46 genes). Five angiogenesis-related genes do not encode PKs targetable by regorafenib at achievable concentrations.

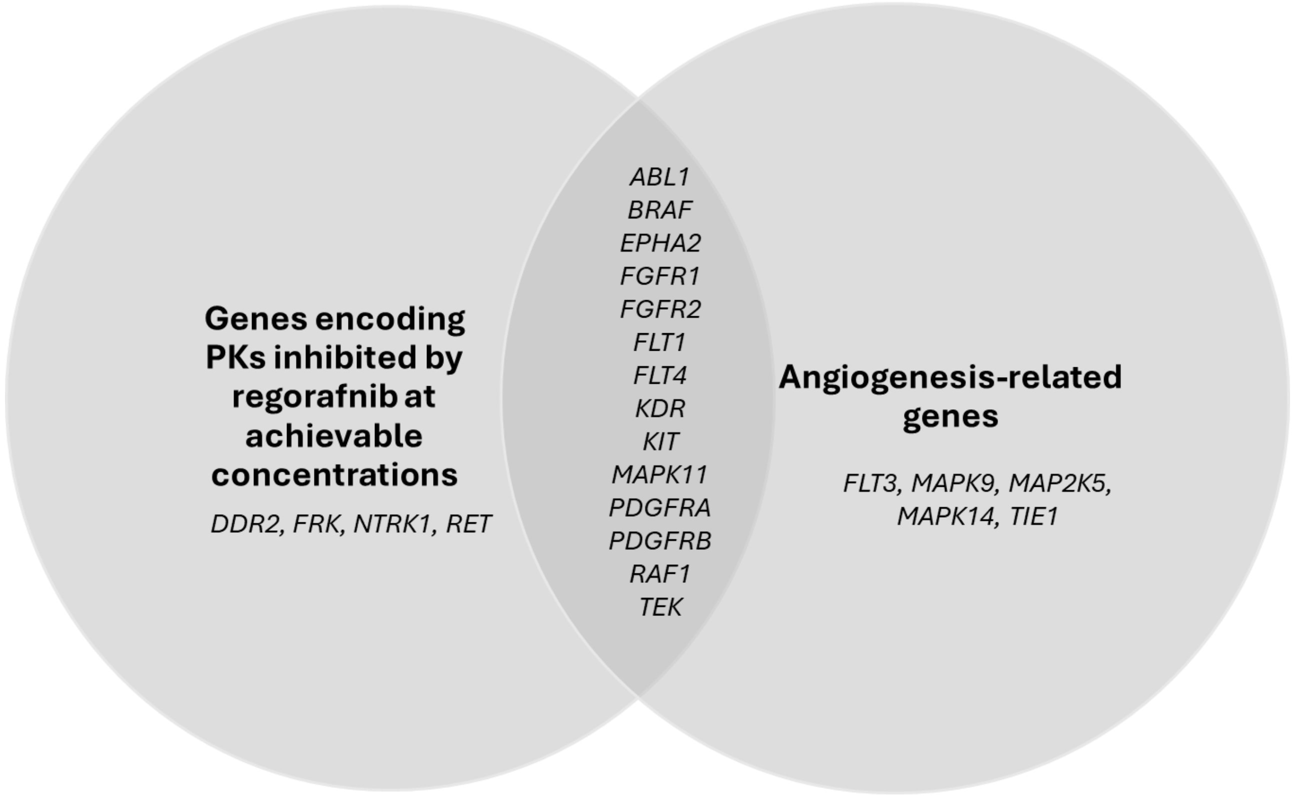
Genes encoding PKs inhibited by regorafnib at achievable concentrations
DDR2, FRK, NTRK1, RET
ABL1
BRAF
EPHA2
FGFR1
FGFR2
FLT1
FLT4
KDR
KIT
MAPK11
PDGFRA
PDGFRB
RAF1
TEK
Angiogenesis-related genes
FLT3, MAPK9, MAP2K5, MAPK14, TIE1
